# The Extraglycemic Effect of SGLT-2is on Mineral and Bone Metabolism and Bone Fracture

**DOI:** 10.3389/fendo.2022.918350

**Published:** 2022-07-07

**Authors:** Bingzi Dong, Ruolin Lv, Jun Wang, Lin Che, Zhongchao Wang, Zhouyang Huai, Yangang Wang, Lili Xu

**Affiliations:** ^1^ Department of Endocrinology and Metabolism, The Affiliated Hospital of Qingdao University, Qingdao, China; ^2^ Department of Nephrology, The Affiliated Hospital of Qingdao University, Qingdao, China; ^3^ Department of Geriatric Medicine, Yantai Yuhuangding Hospital Affiliated Hospital of Qingdao University, Yantai, China

**Keywords:** sodium–glucose cotransporter-2 inhibitor, bone and mineral metabolism, bone turnover, bone fracture risk, type 2 diabetes mellitus

## Abstract

Type 2 diabetes mellitus (T2DM) is a risk factor for osteoporosis. The effects of T2DM and anti-diabetic agents on bone and mineral metabolism have been observed. Sodium–glucose co-transporter 2 inhibitors (SGLT-2is) promote urinary glucose excretion, reduce blood glucose level, and improve the cardiovascular and diabetic nephropathy outcomes. In this review, we focused on the extraglycemic effect and physiological regulation of SGLT-2is on bone and mineral metabolism. SGLT-2is affect the bone turnover, microarchitecture, and bone strength indirectly. Clinical evidence of a meta-analysis showed that SGLT-2is might not increase the risk of bone fracture. The effect of SGLT-2is on bone fracture is controversial, and further investigation from a real-world study is needed. Based on its significant benefit on cardiovascular and chronic kidney disease (CKD) outcomes, SGLT-2is are an outstanding choice. Bone mineral density (BMD) and fracture risk evaluation should be considered for patients with a high risk of bone fracture.

## Background

Type 2 diabetes mellitus (T2DM) is a common metabolic disease that affects individuals worldwide. T2DM is commonly associated with obesity, hypertension, heart failure, hyperuricemia, renal failure, proteinuria, and osteoporosis (1). Patients with T2DM show increased risks of cardiovascular or renal complications, resulting in the main causes of morbidity and mortality (2). Thus, the therapeutic strategy for T2DM concerns with the benefits on cardiovascular–renal complications. Among the glucose-lowering agents, sodium–glucose co-transporter inhibitors (SGLT-2is) are highly recommended for patients with T2DM, especially those with a high cardiovascular or renal risk, due to their remarkable cardiorenal protective effects (3, 4).

T2DM is one of the common risk factors associated with osteoporosis and osteoporotic fracture. As oral anti-diabetic agents, SGLT-2is inhibit the reabsorption of sodium and glucose, reduce the blood glucose level, and cause negative energy balance and body weight loss. However, the effects of SGLT-2is on bone and mineral metabolism and bone fracture risk remain controversial. In this review, we screened publications (**Supplementary Figure S1**) and focused on the extraglycemic effects of SGLT-2is on bone and mineral metabolism and osteoporotic fracture.

## Glucose-Lowering Mechanism

**Figure 1:**
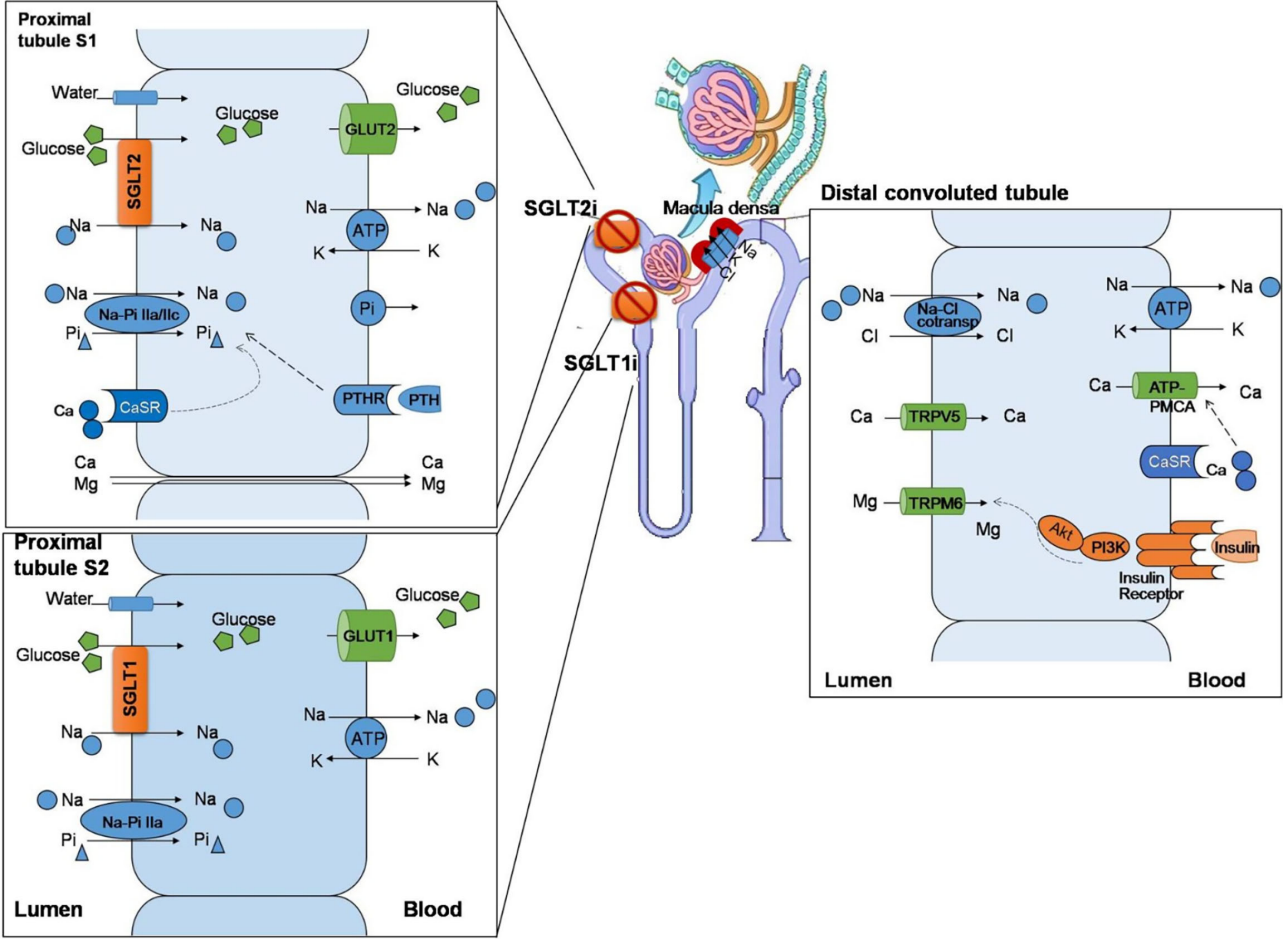
The effect of sodium–glucose co-transporters (SGLTs) on the regulation of mineral ions in renal tubules. The SGLT-1/SGLT-2 co-transporters are mainly expressed in proximal renal tubules, regulating the reabsorption of sodium and glucose. SGLT-2 reabsorbs sodium and glucose from the lumen side of proximal tubules and acts synergistically with glucose transporters (GLUTs) to transport glucose into the blood. In addition, the Na–Pi IIa/c co-transporters are expressed in the lumen side, which synergistically regulate the transport of sodium and phosphorus. Calcium and magnesium pass through intercellular channels. In proximal tubules, the calcium-sensing receptor (CaSR) is located on the lumen side, which senses the concentration of extracellular calcium and synergy with the parathyroid hormone receptor (PTHR), which is stimulated by the parathyroid hormone (PTH), to regulate the transporter function and maintain the hemostasis of calcium/phosphate. Sodium–glucose co-transporter 2 inhibitors (SGLT-2is) increase the excretion of urinary sodium and glucose. The increased sodium delivery in distal convoluted tubules stimulates dense plaques, triggers tubulo-glomerular feedback, and shrinks the afferent glomerular arteriole. In distal convoluted tubules, the insulin receptor is expressed in the blood side. Magnesium reabsorption passes *via* transient receptor potential melastatin type 6 (TRPM6), and the channel activity is suppressed by insulin binding to the insulin receptor *via* the PI3K/Akt signaling pathway. In this way, SGLT-1 and SGLT-2 play an important role in regulating mineral metabolism.

The sodium–glucose linked transporter (SGLT) family has at least six isoforms. At present, SGLT-1 (encoded by the *SGLT1* gene, also known as *SLC5A1*) and SGLT-2 (encoded by the *SGLT2* gene, also known as *SLC5A2*) are known for their critical role in regulating the reabsorption and transportation of sodium and glucose (5). SGLT-1 accounts for most of the dietary glucose uptake by the intestinal tract and the transmembrane transport of glucose and galactose through the brush margin of the small intestine, while SGLT-2 is responsible for the majority of glucose reuptake in the tubular system of the kidney (6). There are two main types of glucose transporters: SGLTs, which co-transport sodium/glucose by an electrochemical gradient across the membrane, and glucose transporters (GLUTs), through which glucose is transported by facilitated diffusion. In proximal tubules of the kidney, SGLT-2 acts synergistically with GLUT-2. SGLT-2 reabsorbs sodium and glucose from the luminal side of proximal tubules. Glucose is transported into the blood *via* GLUT-2 located in the lateral basement membrane. SGLT-2is inhibit the reabsorption of sodium and glucose and increase the excretion of urine. On the other hand, the reduced reabsorption of sodium leads to an increased delivery of sodium to dense plaques. Due to the stimulation of the tubulo-glomerular feedback, as well as the contraction of the afferent glomerular arteriole, glomerular hyperperfusion and hyperfiltration are weakened (**Figure 1**) (7).

Based on these mechanisms, SGLT-2is have been developed as oral anti-diabetic agents. They lower the renal threshold for glucose (RTG) and promote urinary glucose and sodium excretion and osmotic diuresis. SGLT-2is reduce the levels of blood glucose and glycosylated hemoglobin; they also lower the risk of major adverse cardiovascular events, including cardiovascular death, non-fatal myocardial infarction, non-fatal stroke, and risk of hospitalization for heart failure in T2DM patients with established cardiovascular diseases or risk factors (4). In addition, SGLT-2is reduce the progression to albuminuria, decrease the composite outcome of end-stage renal disease, and lower the need for renal replacement therapy or death from renal causes (8). They improve the outcomes of cardiovascular events and the prognosis of heart failure and diabetic kidney disease (DKD). Among the currently commercially available SGLT-2is, empagliflozin, dapagliflozin, ipragliflozin, ertugliflozin, luseogliflozin, and tofogliflozin can selectively inhibit the activity of SGLT-2, while canagliflozin and sotagliflozin also inhibit SGLT-1 (5). In this review, we aimed to provide an update on the regulatory mechanisms and clinical evidence on the bone and mineral effects of SGLT-2is.

## Characteristics of the Bone Metabolism Abnormalities on Diabetic Bone Disease

In patients with T2DM, the bone mineral density (BMD) tends to be increased, but with an increased fracture risk. This phenomenon of a higher BMD may be related to the anabolic effect of hyperinsulinemia. However, an abnormal bone metabolism is characterized by a low bone turnover (9) and increased bone brittleness, leading to the high risk of fracture. The fracture risk could be caused by a decreased bone quality and strength, cortical bone porosity, and impaired bone microarchitecture. The decrease of bone quality in patients with diabetic bone disease (DBD) is accompanied by bone remodeling disorders (10). The pathophysiological mechanism underlying bone fragility in T2DM is complex. Possible mechanisms beyond bone include hyperglycemia, oxidative stress, and tissue-specific accumulation of advanced glycation end products (AGEs) that compromise collagen properties and increase bone marrow adiposity, releasing inflammatory factors and adipokines from visceral fat, chronic inflammatory microenvironment, and potentially altering the function of osteoblasts and osteocytes (11). In diabetic rats, the abnormal accumulation of AGEs in collagen promotes oxidative stress and local inflammatory response and induces the apoptosis of osteoblasts (12). T2DM complications such as retinopathy and cataracts, diabetic neuropathy, and postural hypotension may also contribute to the increased frequency of falls and the consequential bone fractures (13). In aging T2DM patients with fatigue or hypoglycemia, the increased propensity for falls increases the risk of fracture. Furthermore, some anti-diabetic agents may directly or indirectly affect BMD and bone turnover (14). For example, thiazolidinedione, the ligand of proliferator-activated receptor gamma (PPARγ), can directly inhibit the activity of osteoblasts, reduce bone formation and BMD, and increase the risk of fracture (15). Observational studies have shown that exposure to exogenous insulin increases the risk of hip and vertebral fracture, despite the established anabolic effects of insulin on bone matrix synthesis (13).

## Effect of SGLT-2is on Mineral Metabolism

**Figure 2:**
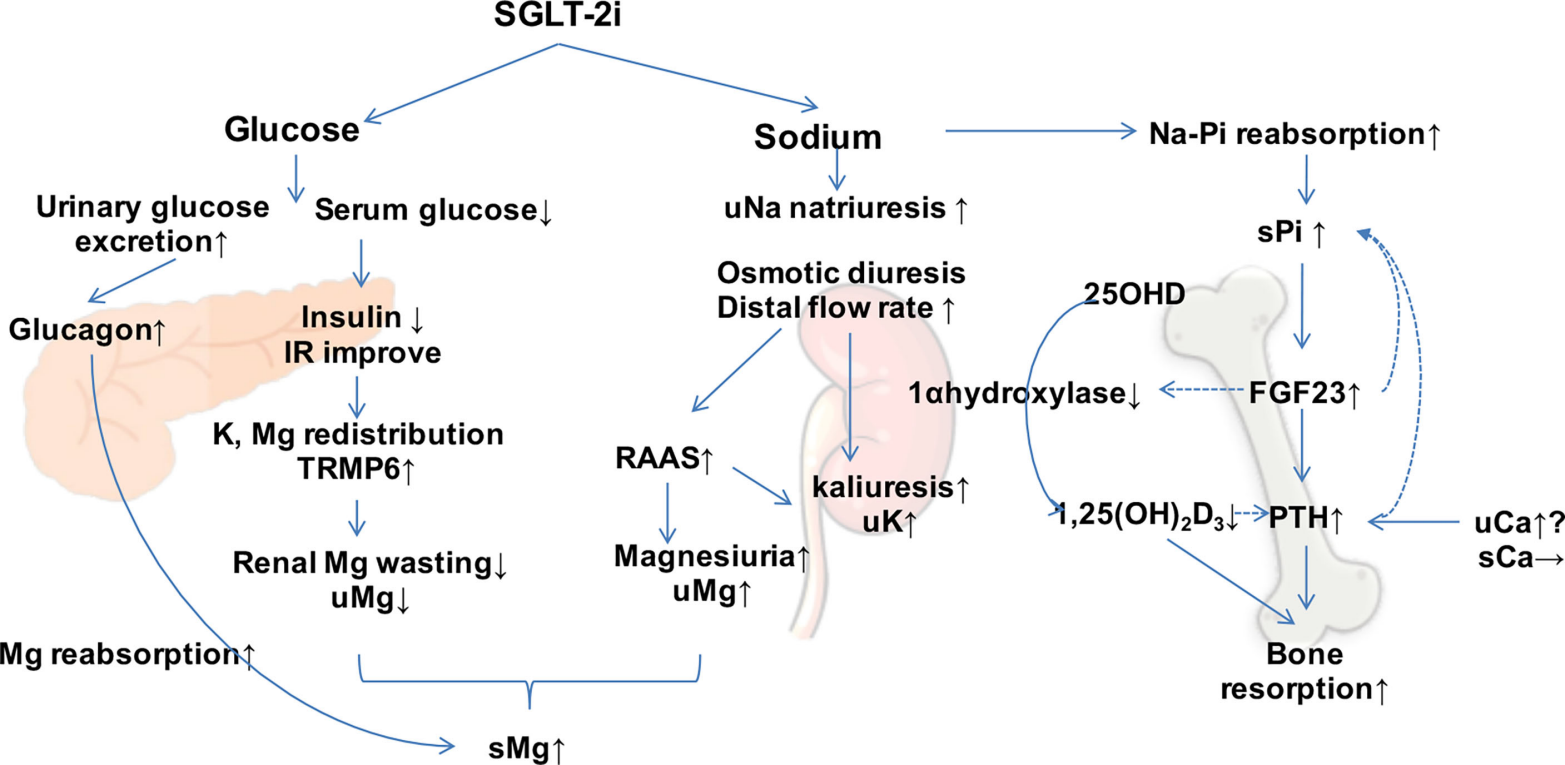
Effect of sodium–glucose co-transporter 2 inhibitors (SGLT-2is) on mineral metabolism. SGLT-2is increase the excretion of urinary glucose and sodium and regulate minerals directly and indirectly. *Dotted arrows* show suppressive effects. *s*, serum; *u*, urinary; *Mg*, magnesium; *Ca*, calcium; *Na*, sodium; *Pi*, phosphorus; *PTH*, parathyroid hormone; *FGF23*, fibroblast growth factor 23; *IR*, insulin resistance; *RAAS*, renin–angiotensin–aldosterone system.

Based on the renal mechanism of SGLT-2is, they may have potential effects on electrolyte homeostasis and bone mineral metabolism. There are multiple interweaving networks of SGLT-2i on mineral and bone metabolism, including affecting the metabolism of electrolytes and calcium/phosphorus (**Figure 1**) and increasing the parathyroid hormone (PTH) and reducing 1,25-(OH)_2_-vitamin D levels (16) (**Figure 2**).

### Sodium

SGLT-2 regulates sodium–glucose reabsorption. In a healthy population, more than 90% of glucose is absorbed through SGLT-2, with less than 10% through SGLT-1. However, the expressions of SGLT-2 and SGLT-1 are upregulated and activated in patients with T2DM, leading to the increased sodium reabsorption in proximal tubules. The excess sodium and glucose accumulate downstream and are reabsorbed supplementarily *via* other sodium transporters such as SGLT-1 and Na^+^/H^+^ exchange protein 3 (NHE3). Thus, sodium uptake is reduced in the dense plaque in order to stimulate the tubulo-glomerular balance feedback regulating system. On the other hand, the decreased synthesis of vasoconstrictor factors, which act on afferent glomerular arterioles, causes an increased renal blood flow and glomerular capillary hydrostatic pressure (17, 18). All of these mechanisms lead to an increased glomerular filtration, which is the pathophysiological basis of increased intraglomerular pressure and DKD. SGLT-2is can reverse these phenomena, regulate the co-transport of sodium–glucose, and modulate the excretion of sodium. In this manner, SGLT-2is play a vital role in reducing the volume load and lowering blood pressure.

One third of sodium is stored in the bone matrix, of which 40% is exchangeable with circulating sodium (19). It has been hypothesized that sodium is released from bones in an attempt to maintain sodium homeostasis, similar to the release of calcium during hypocalcemia (20). Bone resorption increases in the condition called relative hyponatremia, leading to the reduction of BMD and the increase of fracture risk. According to data from the U.S. National Health and Nutrition Examination Survey database, chronic hyponatremia increases the risk of hip fracture (21). In a hyponatremia animal model, a significant decrease in vertebral and cortical BMD was observed. The increased sensitivity of osteoclasts to extracellular sodium stimulates osteoclast differentiation and bone resorption, without a change in the osteoblast number or the bone mineralization rate (21). An *in vitro* study using murine pre-osteoclastic RAW 264.7 cells indicated that a decrease of the medium sodium level could increase the maturation and activity of osteoclasts. A low sodium concentration triggered osteoclast-mediated bone resorption mobilization (22). In addition, SGLT-2is induced hypovolemia, and relative “hyponatremia” may be accompanied by fatigue, weakness, or other psychosomatic and neurological symptoms, prone to coexist with cardiovascular, respiratory, and metabolic chronic diseases. All these factors increase the risk of falls and fractures.

### Magnesium

SGLT-2is can elevate serum magnesium levels. A meta-analysis of 18 randomized controlled trials (RCTs) that involved 15,309 patients with T2DM administered four SGLT-2is (canagliflozin, empagliflozin, dapagliflozin, and ipragliflozin) showed that SGLT-2is can increase serum magnesium levels. Canagliflozin increased serum magnesium in a linear dose-dependent manner (23). Patients with T2DM often exhibit an increased urinary magnesium excretion, which leads to hypomagnesemia (24). Magnesium deficiency might affect BMD. Hypomagnesemia could reduce bone stiffness, increase osteoclast activity, and decrease osteoblast-induced bone formation. It also interferes with PTH and 1,25-(OH)_2_-vitamin D, reduces vitamin D synthesis and activation, and promotes inflammation/oxidative stress, which subsequently causes bone loss (25). Hypomagnesemia promotes inflammation, linking the relationship between chronic inflammation and bone loss (26). In magnesium-deficient rodents, the levels of TNF-α, IL-1, and IL-6 were increased both in serum and the bone marrow microenvironment (27). Furthermore, substance P is highly released during magnesium deficiency. To enhance the secretion of pro-inflammatory cytokines, substance P released on the nerve endings in bones stimulated the activity of osteoclasts (28). On the other hand, increased urinary magnesium may be associated with insulin resistance due to the decreased activity of the transient receptor potential melastatin type 6 (TRPM6) cation channel in distal renal collecting tubules (29). Type 2 obese diabetic rats showed downregulation of TRPM6 channel expression, hypermagneuria, and hypomagnesemia (30).

Clinical studies have suggested that SGLT-2is can increase the serum magnesium levels in patients with T2DM (31, 32). Osmotic diuresis results in a decrease in the extracellular volume load, which leads to a mild increase in blood magnesium levels (33). In addition, SGLT-2is induce elevated glucagon concentration and increase the magnesium reabsorption in distal tubules, also affecting magnesium homeostasis (34). SGLT-2is reduce the circulating insulin levels and improve insulin resistance, possibly leading to an increased TRPM6 activity and a reduced urinary magnesium excretion. Moreover, insulin promotes the transfer of magnesium into cells and interferes with the distribution of intercellular and extracellular magnesium (35). SGLT-2is also increase the concentration of aldosterone by natriuretic function and reduce the volume load, which has a direct effect on the transport of magnesium, causing its increased excretion (36). The complex mechanism of SGLT-2is on magnesium hemostasis has not been fully clarified. The regulatory mechanisms of magnesium ultimately result in a mild increase in serum magnesium levels.

Two-thirds of magnesium is stored in bone tissue. Magnesium directly regulates bone metabolism by modulating bone growth and bone strength. Magnesium deficiency results in a decreased osteoblast function and an increased osteoclast activity (28). An animal model of magnesium deficiency showed reduced trabecular number and thickness. The histological analysis revealed that the bone formation was reduced and the bone resorption was enhanced. On the contrary, the trabecular bone volume, maximum load, and elastic modulus were reduced (37). This may explain the mechanism of SGLT-2is improving bone metabolism by elevating the serum magnesium levels. On the other hand, magnesium indirectly influences bone metabolism by regulating the levels of PTH and 1,25-(OH)_2_-vitamin D. Basic research has suggested that magnesium is involved in improving the regulation of local inflammatory microenvironment and oxidative stress, which may indirectly affect bone metabolic processes (38).

### Calcium

SGLT-2is cause a mild increase in urinary calcium excretion without obvious changes in the serum calcium levels (39). SGLT-2is inhibit the reabsorption of glucose in renal tubules, leading to osmotic diuresis, which increases tubular blood flow and reduces the calcium reabsorption in proximal tubules and the difference of the calcium gradient between tubules and the renal interstitium (40). A high urinary calcium is often observed in congenital transporter dysfunction, such as inherited SGLT-2 deficiency and familial renal glycosuria (41), similar to the pharmacological changes of SGLT-2is. An elevated urinary calcium excretion leads to a feedback increase in the PTH level, which stimulates bone turnover. PTH can increase serum calcium and decrease phosphorus by mobilizing bone osteolysis and enhancing urine phosphorus excretion. Patients with T2DM treated with canagliflozin showed reduced BMD in the hip. At pharmacological doses, canagliflozin did not significantly elevate urinary calcium or magnesium excretion. This might be due to the incomplete blockage of the transporter and compensation by SGLT-1, which increased the reabsorption of calcium from the distal nephron (42). SGLT-2is stimulate an increased urinary calcium excretion and increased renal phosphate reabsorption, leading to a decrease in the levels of active vitamin D, further reducing calcium absorption through the gastrointestinal tract. This possibly affects bone mineralization. However, more large-scale clinical trials are needed to confirm the link between calcium hemostasis affected by SGLT-2is.

### Phosphorus

SGLT-2is inhibit sodium–glucose co-transport and reabsorption. Sodium-dependent sodium–phosphate transporters IIa (Na–Pi IIa, encoded by *SLC24A1*) and IIc (Na–Pi IIc, encoded by *SLC34A3*) are activated, and phosphate reabsorption is increased in proximal tubules to maintain the sodium concentration gradient in the tubule (43). In a single-blind, randomized crossover study, 25 healthy volunteers were observed to have a slight but significant increase in serum phosphorus levels after the administration of canagliflozin. On the other hand, the levels of serum fibroblast growth factor 23 (FGF23) and PTH were elevated, while that of 1,25-(OH)_2_-vitamin D showed a continuous reduction (44). A sustained increase in PTH enhances bone resorption and increases the risk of bone fracture. Similarly, increased levels of FGF23 are associated with bone metabolic disorders. Reduced 1,25-(OH)_2_-vitamin D levels diminished the absorption of calcium from the intestine and impaired bone calcification (43). FGF23 is an important hormone regulating the excretion of urinary phosphorus. However, it has adverse extrarenal outcomes such as heart failure and cardiovascular events. FGF23 is secreted by mature osteocytes to regulate intestinal phosphorus uptake and urinary excretion simultaneously and to maintain phosphorus hemostasis. Hyperphosphatemia stimulates the synthesis of FGF23 by osteocytes. FGF23 inhibits the reabsorption of phosphorus in proximal tubules, promotes the excretion of urinary phosphorus by downregulating the expression of the Na–Pi IIa transporter, and reduces the release of phosphate from the bone by inhibiting the secretion of PTH. Furthermore, FGF23 reduces the conversion of 25-hydroxyvitamin D (25-OHD) to active 1,25-(OH)_2_-vitamin D by inhibiting the activity of 1-α-hydroxylase, which results in a reduced phosphate absorption through the intestine (45). In a diabetic mouse model, canagliflozin induced a significant decrease in the messenger RNA (mRNA) expression of 25-OHD–1alpha hydroxylase (encoded by *CYP27B1*) (46). In turn, a low vitamin D level feedback stimulates the release of PTH and decreases the absorption of calcium through the intestine. It has been reported that the application of canagliflozin is associated with elevated blood phosphorus levels in diabetic patients (47). Treatment with dapagliflozin results in elevated serum phosphorus levels, accompanied by significant increases in the levels of PTH and serum FGF23 (48). SGLT-2is stimulate the reabsorption of renal phosphorus and the excretion of urinary calcium, leading to a feedback increase in the levels of FGF23 and PTH and a decrease in active vitamin D. However, the increased levels of PTH and FGF23 further promote the excretion of phosphorus. In T2DM patients with chronic renal insufficiency and renal bone disease, hyperphosphatemia causes dysfunction of the FGF23–1,25-(OH)_2_-vitamin D–PTH axis and exacerbates the risk of fracture (49) (**Figure 2**).

## Effect of SGLT-2is on Bone Turnover

Bone turnover determines the dynamic balance between osteoclast-induced bone resorption and osteoblast-mediated bone formation, which is the key factor determining bone quality. Patients with DBD show bone metabolic abnormalities with features of low bone turnover (50). SGLT-1 and SGLT-2 are not expressed in mouse cranial osteoblasts, bone marrow macrophages, or mature osteoclasts (51). Therefore, the effects of SGLT-2is on bone metabolism and bone turnover may be indirect. The effect on bone remodeling differs from that of SGLT-2is. Canagliflozin increases the level of type I collagen carboxyl-terminal peptide β special sequence (β-CTX), a marker of bone resorption. At the same time, the bone formation marker osteocalcin was enhanced significantly (52). However, dapagliflozin and empagliflozin showed no significant effect on type I procollagen amino-terminal propeptide (PINP) or β-CTX (53). A subgroup analysis of a randomized controlled study was carried out to evaluate the effects of ipragliflozin on bone in Japanese patients with T2DM (baseline body mass index, ≥22 kg/m^2^; hemoglobin A1c, 7%–10%). The results showed that the SGLT-2i ipragliflozin had a negative effect on both bone and muscle content. The levels of tartrate-resistant acid phosphatase 5b (TRACP-5b), a bone resorption marker, increased from baseline between 12 and 24 weeks in the ipragliflozin group. The levels of bone-specific alkali phosphatase (BAP), a bone formation marker, were not significantly changed, suggesting that ipragliflozin promoted bone resorption, but not bone formation. However, there were no significant changes in vertebral BMD over 24 weeks. A long-term study is required for further understanding of the effects of ipragliflozin on BMD and bone remodeling (54). In addition, weight loss induced by SGLT-2is may also affect BMD and bone turnover. An RCT study showed that elderly obese patients exhibited reduced hip BMD after weight loss. On the other hand, the bone formation marker osteocalcin and the bone resorption marker β-CTX were simultaneously elevated, suggesting that body weight loss may enhance bone turnover and lead to negative bone regulation (55). Adiponectin, an anti-inflammatory cytokine, is upregulated after fat loss, which also modulates the osteoclast response activity by inhibiting the ratio of the receptor activator of NFκB ligand to osteoprotegerin (RANKL/OPG) (56). The activation of adiponectin receptors in osteoblasts inhibits sclerostin, the Wnt pathway inhibitor, and promotes osteoblast differentiation and maturation (57). The mechanism of the effect of SGLT-2is on bone turnover is complex and inconclusive. Firm evidence from larger-scale clinical trials is still needed.

## Effect of SGLT-2is on Bone Microarchitecture and Bone Strength

Microstructure abnormalities in vertebral and cortical bone can be observed in postmenopausal patients with T2DM. The classic feature is characterized as an increased porosity in cortical bone (58), which disturbs bone strength and increases the risk of fracture. In a clinical study involving 716 patients with T2DM who received a 52-week administration of canagliflozin, there were no significant differences in vertebral and femoral neck BMD and bone strength compared to the placebo group (52). Japanese researchers recruited patients with T2DM and investigated whether the SGLT-2i luseogliflozin, with a higher SGLT-2 selectivity, could affect the bone microarchitecture. The effect of luseogliflozin on bone metabolism was evaluated using high-resolution peripheral quantitative computed tomography (HR-pqCT), which was expected to provide direct *in vivo* morphometric information about the bone microarchitecture (59). Due to the limitations of the specimen and the detection method, further clinical investigations of the effects of SGLT-2is on bone matrix mineralization, collagen synthesis, and biomechanics of the bone microstructure are expected in the future. Subsequently, the effects of SGLT-2is on bone microstructure and bone strength will be elucidated from molecular mechanism and animal studies to clinical outcomes in depth.

## Effect of SGLT-2is on Bone in Animal Models

In animal models of diabetes, deficits in the trabecular bone microarchitecture can be observed, including decreased trabecular bone volume (BV/TV), trabecular number (Tb.N), and trabecular thickness (Tb.Th), with increased trabecular spacing (Tb.Sp) and changes in the trabecular shape (SMI). In addition, diabetic mice exhibited deficits in cortical bone content combined with increased cortical porosity and decreased material properties, bending strength, and toughness. The structural properties including structure strength and stiffness were reduced in mice with diabetes mellitus (DM). All these factors contribute to bone fracture (39). *Slc5a2* mutant mice with SGLT2 deletion showed a reduction in cortical BMD, mild defects in cortical bone mineralization, and normal bone remodeling (60). In animal studies (**Table 1**), canagliflozin showed negative bone effects, including reduced femoral BV/TV and Tb.N and increased Tb.Sp. However, its administration combined with insulin prevented DM-related deterioration in the bone microarchitecture and bone strength (39, 61). In T2DM mouse models, canagliflozin partially improved the cortical and tracecular bone deficits (62). Dapagliflozin ameliorated bone tissue material properties, bone matrix strength, and matrix biomechanics (maximum load, indentation modulus, and hardness) (63).

**Table 1:** The effect of SGLT-2 and inhibitors on bone and mineral metabolism from animal studies.

Reference	Mice strain	Agents	Blood glucose and metabolic parameters	Effect on bone	Mineral metabolism
Thrailkill et al. 2016 (39)	DBA2J +STZ T1DM model	Canagliflozin	Canagliflozin decrease BG level	Canagliflozin reduce femoral BV/TV, Tb.N, increase Tb.Sp	uCa, FGF23 increased, maybe compensatory hyperparathyroidism to uCa, reduce CYP27B1 mRNA
Thrailkill et al. 2017 (61)	DBA2J +STZ T1DM model	Canagliflozin vs/+ insulin	Achieved normal BG of DM mice	Canagliflozin combination with insulin prevent DM-related deterioration in bone microarchitecture and bone strength	uCa, FGF23 increased
Trailkill et al. 2020 (62)	TallyHO mice T2DM model	Canagliflozin	ND	Canagliflozin partically corrected the cortical bone deficits (normalized Ct.Ar, but low Ct.Th and high medullary volume persisted). Improvement tendency of trabecular parameters (Tb.Th, BV/TV, SMI). Not improved material strength (yield stress, ultimate stress), but increased yield force.	Hypercalciuria, hyperphosphaturia, urinary mineral loss
Mieczkowska A et al. (63)	Swiss mice T2DM model+STZ	Dapagliflozin vs liraglutide	Both reduced FBG and insulin resistance	Short-term treatment did not restore bone strength and microarchitecture. Dapagliflozin ameliorated bone tissue material properties (increase mineral maturity ratio, decrease crystal size index), bone matrix strength (collagen crosslinks and glycation) and matrix biomechanics (maximum load, indentation modulus, hardness).	ND
Suzuki et al. (64)	HFD Wistar rat, KKAy mice T2DM model	Togogliflozin	Reduced glucose, body weight gain and adipose tissue, adipocyte size, inflammatory infiltration	no change of bone mass	ND
Gerber et al. (60)	SP mice with scl5a2 deletion	gene mutation	SP mice (Slgt2 del) showed hyperglucosuria	SP mice (Slgt2 del) showed reduction in cortical BMD, mild defects in cortical bone mineralization and normal bone remodeling.	Hyperphosphuria but normal serum phosphate, without hypercalciuria

## Effect of SGLT-2is on the Risk of Fracture

The effects of SGLT-2is on the risk of fracture still remain controversial. They may vary across different agents (**Tables 2** and **3**). In the Canagliflozin Cardiovascular Assessment (CANVAS) Study, bone fractures and a decreased total hip BMD in the canagliflozin group were significantly higher than those in the placebo group. However, no increased risk was observed in the CANVAS—Renal (CANVAS-R) Study (hazard ratio, HR = 0.86) (65). In the Dapagliflozin Effect on Cardiovascular Events (DECLARE) Study including 17,160 patients with T2DM, the risk of fracture in the dapagliflozin group was comparable to that in the placebo group (66). Similarly, the EMPA-REG-OUTCOME [BI 10773 (Empagliflozin) Cardiovascular Outcome Event Trial in Type 2 Diabetes Mellitus Patients] Study showed that the risk of fracture was 1.4%–1.7% in the empagliflozin group at different doses (10 or 25 mg/day), both of which were comparable to those in the placebo group (67). In the VERTIS CV (Evaluation of Ertugliflozin Efficacy and Safety Cardiovascular Outcomes Trial) Study, the administration of ertugliflozin did not significantly increase the risk of fracture (68). Among the presently available SGLT-2is, canagliflozin has been reported to be associated with an increased risk of fracture (69).

**Table 2:** Clinical trials information about SGLT2is.

Drug	No.*	Dose	Total patients and (N-Drug/Placebo)	Follow-up time	Trials and numbers	Main Responsible Company
Dapagliflozin	1	10mg/Placebo	17160 (8582/8578)	5.2y-6y	DECLARE-TIMI 58 (NCT01730534)	AstraZeneca
	2	10mg/Placebo	4304 (2152/2152)	38.2m	DAPA-CKD (NCT03036150)	AstraZeneca
	3	10mg/Placebo	4744 (2373/2371)	27.8m	DAPA-HF (NCT03036124)	AstraZeneca
	4	10mg/Placebo	321 (160/161)	24w	DERIVE (NCT02413398)	AstraZeneca
Canagliflozin	5	100mg/300mg/Placebo	4330 (1445/1443/1442)	4y-8y	CANVAS (NCT01032629)	Janssen Research &Development
	6	100mg (the first 13 weeks), 300mg (after 13 weeks)/Placebo	5812 (2907/2905)	3y	CANVAS-R (NCT01989754)	Janssen Research &Development
	7	Pressure test/CT/CTA (CAG)	618 (309/309)	48m-60m	CREDENCE (NCT02173275)	Weill Medical College of Cornell University
	8		12227 (effect analysis 11675)	3y	SAPPHIRE (JapicCTI-153048)	
Empagliflozin	9	10mg/25mg/Placebo	7020 (2345/2342/2333)	4.6y	EMPA-REG OUTCOME (NCT01131676)	Boehringer Ingelheim, Eli Lilly
	10	10mg/Placebo	3730 (1867/1863)	1040d	EMPEROR-Reduced (NCT03057977)	Boehringer Ingelheim, Eli Lilly
Ipragliflozin L-Proline	11	50mg/Placebo	8505	1y	STELLA-ELDER (NCT02297620)	Astellas Pharma Inc
	12	50mg/Placebo	11424 (safety analysis 11051, effect analysis 8763)	3y	STELLA-LONG TERM (NCT02479399)	Astellas Pharma Inc
	13	50mg/DMBG 1000-1500mg qd		24w	PRIME-V study (UMIN 000015170)	Astellas Pharma Inc
Tofogliflozin	14	5mg/Placebo	6897 (safety analysis 6711, effect analysis 6451)	3y	J-STEP/ LT (PMID 32597517)	Kowa Company, Ltd. And Sanofi K. K.
Luseogliflozin	15	2.5mg/Placebo	43	52w	LIGHT (UMIN000015112)	Taisho Toyama Pharmaceutical Co., Ltd. and Novo Nordisk Pharma
Ertugliflozin;MK-8835	16	5mg/15mg/Placebo	8246 (2752/2747/2747)	6y	VERTIS CV (NCT01986881)	Merck Sharp & Dohme Corp.
Bexagliflozin	17	20mg/Placebo	312 (157/155)	24w	PMID 31101403	TheracosSub,LLC
	18	Bexagliflozin 20mg/Sitagliptin 100mg	384 (191/193)	24w	PMID 31161692/34292100	Theracos Sub, LLC
	19	20mg/Placebo	1700 (1133/567)	24w-48w	RCT (NCT02558296)	Theracos Sub, LLC
LIK-066 (Licogliflozin)	20	LIK066 2.5mg/10mg/50mg/EMPA 25mg/Placebo	124 (15/16/30/30/33)	36w	RCT (NCT03320941)	Novartis Pharmaceuticals
Henagliflozin Proline/SHR3824	21	5mg/10mg/Placebo	450	24w-52w	RCT (NCT04390295)	Jiangsu HengRui Medicine Co., Ltd.
Remogliflozin etabonate	22	100mg/250mg bid/ dapagliflozin 10mg	612 (167/175/103)	24w	RCT (CTRI/2017/07/009121)	GSK

*Table 1 and **Table 2** correspondbycolumnof No.

**Table 3:** Clinical trials information about SGLT2is and bone fracture risk.

No.*	Drug	Trials and numbers	Fracture percentage	Result	Primary outcome	Second outcome
1	Dapagliflozin	DECLARE-TIMI 58 (NCT01730534)	5.3/5.1	total complete 8574/8569, Acetabulum fracture 3:1 (0.03:0.01) Ankle fracture 19:14 (0.22:0.16) Avulsion fracture 0:1 (0.00:0.01) Cervical vertebral fracture 1:4 (0.01:0.05) Clavicle fracture 1:1 (0.01:0.01) Compression fracture 2:0 (0.02:0.00) Costal cartilage fracture 1:1 (0.01:0.01) Facial bones fracture 10:4 (0.12:0.05) **Femoral neck fracture 5:9 (0.06:0.11) Femur fracture 16:18 (0.19:0.21)** Fibula fracture 5:4 (0.06:0.05) Foot fracture 2:5 (0.02:0.06) Sacrum fracture 3:0 (0.03:0.00) **Hip fracture 13:15 (0.15:0.18)** Humerus fracture 11:20 (0.13:0.23) Ilium fracture 0:1 (0.00:0.01) Limb fracture 1:1 (0.01:0.01) Lower limb fracture 5:9 (0.06:0.11) **Lumbar vertebral fracture 5:6 (0.06:0.07**) Multiple fracture s1:0 (0.01:0.00) Patella fracture 3:1 (0.03:0.01) Plevic fracture 3:4 (0.03:0.05) Periprosthetic fracture 1:0 (0.01:0.00) Rib fracture 12:15 (0.14:0.18) Skull fracture 2:6 (0.02:0.07) Skull fracture d base1:1 (0.01:0.01) **Spinal Compression fracture 12:4 (0.14:0.05)** Sternal fracture 2:4 (0.02:0.05) Thoracic vertebral fracture 3:6 (0.03:0.07) Tibia fracture 12:5 (0.14:0.06) Traumatic fracture 1:1 (0.01:0.01) Ulna fracture 3:3 (0.03:0.04) Upper limb fracture 8:4 (0.09:0.05) Wrist fracture 6:5 (0.07:0.06)	1. CV Death, MI or Ischemic Stroke. 2. CV Death or Hospitalization Due to Heart Failure.	1.Subjects Confirmed Sustained ≥40%, Decrease in eGFR to <60 ml/min/1.73m^2^ and/or ESRD and/or Renal or CV Death. 2. All-cause Mortality.
2		DAPA-CKD (NCT03036150)		total complete 2149/2149, Ankle fracture 3:2 (0.14:0.09) Cervical vertebral fracture 2:1 (0.09:0.05) **Femoral neck fracture 2:3 (0.09:0.14) Femur fracture 7:4 (0.33:0.19)** Foot fracture 1:1 (0.05:0.05) Forearm fracture 0:1 (0.00:0.05) Hand fracture 1:2 (0.05:0.09) **Hip fracture 2:2 (0.09:0.09)** Humerus fracture 1:1 (0.05:0.05) Lower limb fracture 3:0 (0.14:0.00) Multiple fracture s1:2 (0.05:0.09) Patella fracture 1:1 (0.05:0.05) Plevic fracture 1:0 (0.05:0.00) Periprosthetic fracture 0:1 (0.00:0.05) Rib fracture 5:2 (0.23:0.09) Scalpula fracture 2:0 (0.09:0.00) **Spinal Compression fracture 2:1 (0.09:0.05) Spinal fracture 3:1 (0.14:0.05)** Tibia fracture 5:1 (0.23:0.05) Upper limb fracture 1:2 (0.05:0.09) Wrist fracture 0:1 (0.00:0.05)	Time to the First Occurrence of Any of the Components of the Composite: ≥50% Sustained Decline in eGFR or Reaching ESRD or CV Death or Renal Death.	1.Time to the first occurrence of composite: ≥50% Sustained Decline in eGFR or Reaching ESRD or Renal Death. 2. CV Death or Hospitalization for Heart Failure. 3. Death From Any Cause.
3		DAPA-HF (NCT03036124)	2.1/2.1	total complete 2368/2368, **Ankle fracture 3:2 (0.14:0.09) Cervical vertebral fracture 2:1 (0.09:0.05) Femoral neck fracture 2:3 (0.09:0.14) Femur fracture 7:4 (0.33:0.19)** Foot fracture 1:1 (0.05:0.05) Forearm fracture 0:1 (0.00:0.05) Hand fracture 1:2 (0.05:0.09) **Hip fracture 2:2 (0.09:0.09)** Humerus fracture 1:1 (0.05:0.05) Lower limb fracture 3:0 (0.14:0.00) Multiple fracture s1:2 (0.05:0.09) Patella fracture 1:1 (0.05:0.05) Plevic fracture 1:0 (0.05:0.00) Rib fracture 5:2 (0.23:0.09) Scalpula fracture 2:0 (0.09:0.00) Tibia fracture 5:1 (0.23:0.05) Upper limb fracture 1:2 (0.05:0.09) Wrist fracture 0:1 (0.00:0.05) Back pain44:50 (2.05:2.33)	CV Death, Hospitalization Due to Heart Failure or Urgent Visit Due to Heart Failure.	1. CV Death or Hospitalization Due to Heart Failure. 2. Recurrent Hospitalizations Due to Heart Failure and CV Death. 3.Change From Baseline in the KCCQ Total Symptom Score. 4.≥50% Sustained Decline in eGFR, ESRD or Renal Death. 5. All-cause Mortality.
4		DERIVE (NCT02413398)	0/0	No adverse events of fracture were observed in two groups.	Adjusted Mean Change in HbA1c at week 24	1. Percent Change in Total Body Weight at Week 24. 2. Change in Fasting Plasma Glucose (FPG) at Week 24. 3. Change in Seated Systolic Blood Pressure (SBP) at Week 24.
5	Canagliflozin	CANVAS (NCT01032629)	15.40/11.93 (16.3/16.4/10.8, Fall-related incidence 1.9%/3.3%/1.5%	total complete 1445/1441/1441, Acetabulum fracture 0:0:1 Cervical vertebral fracture 0:1:0 Clavicle fracture 1:0:0 Facial bones fracture 1:0:0 **Femoral neck fracture 4:4:0 Femur fracture 1:7:1** Fibula fracture 2:0:0 Foot fracture 0:2:0 **Hip fracture 4:7:3** Humerus fracture 4:8:2 Lower limb fracture 1:0:2 **Lumbar vertebral fracture 1:1:0** Patella fracture 2:1:0 Plevic fracture 0:1:1 Pubis fracture 1:1:0 Rib fracture 3:8:1 Skull fracture 0:3:0 Skull fracture d base1:2:0 **Spinal Compression fracture 0:2:1 Spinal fracture 0:1:0** Sternal fracture 2:0:0 **Thoracic vertebral fracture 2:0:0** Tibia fracture 4:1:0 Ulna fracture 1:1:0 Upper limb fracture 2:1:3 Wrist fracture 1:0:0	Major Adverse Cardiovascular Events (MACE) Composite of Cardiovascular (CV) Death, Non-Fatal MI, and Non-Fatal Stroke.	1.Change in Homeostasis Model Assessment 2 Steady-State Beta-Cell Function (HOMA2-%B), Proinsulin/Insulin (PI/I) Ratio, Urinary Albumin/Creatinine Ratio, eGFR, HbA1c, FPG, SBP,lipid profile. 2.Percentage of Participants With Progression of Albuminuria
6		CANVAS-R (NCT01989754)		total complete 2904/2903, Clavicle fracture 1:0 (0.03:0.00) Epiphysis fracture 1:0 (0.03:0.00) Facial bones fracture 3:1 (0.10:0.03) **Femoral neck fracture 0:2 (0.00:0.07) Femur fracture 4:6 (0.14:0.21)** Fibula fracture 2:1 (0.07:0.03) Foot fracture 1:1 (0.03:0.03) Coccyx fracture 1:0 (0.03:0.00) Hand fracture 2:1 (0.07:0.03) **Hip fracture 3:5 (0.10:0.17)** Humerus fracture 3:2 (0.10:0.07) Jaw fracture 1:0 (0.03:0.00) **Lumbar vertebralfracture 1:1 (0.03:0.03)** Patella fracture 1:2 (0.03:0.07) Pubis fracture 1:0 (0.03:0.00) Rib fracture 4:4 (0.14:0.14) **Spinal fracture 2:0 (0.07:0.00) Thoracic vertebral fracture 0:1 (0.00:0.03)** Tibia fracture 4:1 (0.14:0.03) Traumatic fracture 2:0 (0.07:0.00) Ulna fracture 2:1 (0.07:0.03) Upper limb fracture 1:2 (0.03;0.07) Wrist fracture 0:1 (0.00:0.03) Pathological fracture 1:0 (0.03:0.00)	Progression of Albuminuria.	1.Composite of CV death events or hospitalization for heart failure. 2.CV death.
7		CREDENCE (NCT02173275)	11.8/12.2	date to be released	Diagnostic accuracy of vessel territory-specific ischemia of an integrated stenosis-APC-FFRCT measure by CT.	1.Individual comparisons of APCs or FFRCT to MPI vessel-specific perfusion deficits or reduced MBF. 2.Post-PCI FFR prediction by FFRCT "virtual stenting".
8		SAPPHIRE (JapicCTI-153048)	7 (0.056%)	Ten patients (0.08%) was reported adverse response about bone metabolism, seven of them suffered fracture .	1. ADR. 2.HbA1c	1. ADR. 2. FBG
9	Empagliflozin	EMPA-REG OUTCOME (NCT01131676)	3.8/3.9; 3.1/3.5 (<65y); 4.6/4.3 (65y-75y); 5.2/4.8 (≥75y) (1.9/1.8)	total complete 2345/2342/2333, Acetabulum fracture 1:0:0 (0.04:0.00:0.00) Ankle fracture 4:2:2 (0.17:0.09:0.09) Avulsion fracture 1:0:0 (0.04:0.00:0.00) Clavicle fracture 0:1:1 (0.00:0.04:0.04) Facial bones fracture 0:1:2 (0.00:0.04:0.09) **Femoral neck fracture 1:2:1 (0.04:0.09:0.04) Femur fracture 0:2:3 (0.00:0.09:0.13)** Fibula fracture 1:0:2 (0.04:0.00:0.09) Foot fracture 0:0:1 (0.00:0.00:0.04) Hand fracture 1:0:1 (0.04:0.00:0.04) **Hip fracture 1:7:2 (0.04:0.30:0.09)** Humerus fracture 2:3:0 (0.09:0.13:0.00) Lower limb fracture 1:0:2 (0.04:0.00:0.09) Multiple fracture s0:0:1 (0.00:0.00:0.04) Open fracture 1:0:1 (0.04:0.00:0.04) Plevic fracture 0:1:0 (0.00:0.04:0.00) Periprosthetic fracture 1:0:1 (0.04:0.00:0.04) Pubis fracture 0:1:0 (0.00:0.04:0.00) Rib fracture 4:4:6 (0.17:0.17:0.26) **Spinal Compression fracture 0:3:1 (0.00:0.13:0.04) Spinal fracture 0:1:0 (0.00:0.04:0.00) Thoracic vertebral fracture 0:0:1 (0.00:0.00:0.04)** Tibia fracture 0:2:7 (0.00:0.09:0.30) Ulna fracture 1:1:0 (0.04:0.04:0.00) Upper limb fracture 0:2:0 (0.00:0.09:0.00) Wrist fracture 1:1:0 (0.04:0.04:0.00)	Time to the First Occurrence of Any of the Following Adjudicated Components of the Primary Composite Endpoint (3-point MACE): CV Death, Non-fatal MI, and Non-fatal Stroke.	1.Percentage of All Events Adjudicated (4-point MACE) : CV Death, Non-fatal MI, Non-fatal Stroke and Hospitalization for Unstable Angina Pectoris. 2.Percentage of Participants With Silent MI, Heart Failure Requiring Hospitalisation, New Onset Albuminuria, New Onset Macroalbuminuria, the Composite Microvascular Outcome.
10		EMPEROR-Reduced (NCT03057977)		Both group has 1863 patients completed. Ankle fracture 2:3 (0.11:0.16) Avulsion fracture 2:0 (0.11:0.00) Costal cartilage fracture 1:0 (0.05:0.00) **Femoral neck fracture 1:2 (0.05:0.11) Femur fracture 5:3 (0.27:0.16)** Fibula fracture 0:1 (0.00:0.05) **Hip fracture 2:3 (0.11:0.16)** Humerus fracture 2:1 (0.11:0.05) Jaw 1:0 (0.05:0.00) **Lumbar vertebral fracture 2:1 (0.11:0.05)** Plevic fracture 1:3 (0.05:0.16) Rib fracture 0:2 (0.00:0.11) **Spinal Compression fracture 1:0 (0.05:0.00)** Tibia fracture 0:1 (0.00:0.05) Traumatic fracture 6:0 (0.32:0.00) Upper limb fracture 0:1 (0.00:0.05) Wrist fracture 0:1 (0.00:0.05)	Time to the First Event of Adjudicated CV Death or Adjudicated Hospitalisation for Heart Failure.	1.Occurrence of Adjudicated Hospitalisation for Heart Failure. 2.eGFR (CKD-EPI) cr Slope of Change. 3.Time to First Event in Composite Renal Endpoint: Chronic Dialysis, Renal Transplant or Sustained Reduction of eGFR (CKD-EPI) cr. 4.Time to First Adjudicated Hospitalisation for Heart Failure, Adjudicated CV Death, All-cause Mortality, Onset of Diabetes Mellitus. 5.Change in KCCQ Clinical Summary Score at Week 52. 6.Number of All-cause Hospitalizations.
11	Ipragliflozin L-Proline	STELLA-ELDER (NCT02297620)	2 (0.02%)	Two patients suffered fracture , one was femoral neck, the other was radius fracture .	Specify incidence rates of adverse drug reactions associated with a decrease in body fluids and their risk factors.	occurrence of urinary tract infection, adverse drug reactions in patients at a high risk
12		STELLA-LONG TERM (NCT02479399)	4 (0.04%)	Four patients suffered fracture in drug group	Incidence of cardiovascular adverse events, malignant tumor.	Safety developed by adverse events and laboratory tests
13		PRIME-V study (UMIN 000015170)		After 24 weeks of treatment, the changes in bone mineral density of the fourth lumbar vertebra, handgrip strength and abdominal cross-sectional muscle area were not significantly different between the two groups. However, TRACP-5b levels increased in patients treated with ipragliflozin compared with metformin (median 11.94% vs 10.30%, P<0.0001), suggesting that ipragliflozin may promote bone resorption.	Change in visceral fat area as measured by computed tomography after 24 weeks.	Effects on glucose and lipid metabolism.
14	Tofogliflozin	J-STEP/ LT (PMID 32597517)	2 (0.03%)	Two fracture reported	Recorded safety in terms of adverse drug reactions (ADRs)	Effectiveness in terms of changes in HbA1c and bodyweight
15	Luseogliflozin	LIGHT (UMIN000015112)		date to be released	Metabolic profile changes correlate with body component changes.	ADR and bone mineral content (BMC) change.
16	Ertugliflozin;MK-8835	VERTIS CV (NCT01986881)	2.5%/0.0%/0.6%	total complete 2746/2747/2745, Ankle fracture 3:2:2 (0.11:0.07:0.07) Avulsion fracture 0:1:0 (0.00:0.04:0.00) Cervical vertebralfracture 0:0:1 (0.00:0.00:0.04) Comminuted fracture 1:0:0 (0.04:0.00:0.00) Epiphyseal fracture 0:0:1 (0.00:0.00:0.04) Facial bones fracture 2:1:0 (0.07:0.04:0.00) **Femoral neck fracture 3:3:2 (0.11:0.11:0.07) Femur fracture 6:2:5 (0.22:0.07:0.18)** Fibula fracture 1:1:0 (0.04:0.04:0.00) Foot fracture 1:1:0 (0.04:0.04:0.00) Forearm fracture 0:0:1 (0.00:0.00:0.04) Hand fracture 0:1:0 (0.00:0.04:0.00) **Hip fracture 4:3:4 (0.15:0.11:0.15)** Humerus fracture 4:2:6 (0.15:0.07:0.22) Jaw1:0:0 (0.04:0.00:0.00) Lower limb fracture 0:0:3 (0.00:0.00:0.11) **Lumbar vertebral fracture 2:1:1 (0.07:0.04:0.04)** Patella fracture 0:1:0 (0.00:0.04:0.00) Plevic fracture 0:1:0 (0.00:0.04:0.00) Radius fracture 1:3:3 (0.04:0.11:0.11) Rib fracture 2:5:1 (0.07:0.18:0.04) Skull fracture 2:0:0 (0.07:0.00:0.00) Skull fracture d base0:1:0 (0.00:0.04:0.00) **Spinal Compression fracture 1:5:0 (0.04:0.18:0.00) Spinal fracture 1:1:1 (0.04:0.04:0.04)**	1.Time to First Occurrence of MACE (Cardiovascular Death, Non-fatal MI or Non-fatal Stroke). 2.Baseline HbA1c in different conditions.	Time to Occurrence of CV Death or Hospitalization for Heart Failure, First Occurrence of the Renal Composite, First Occurrence of MACE Plus (Composite Endpoint of CV Death, Non-fatal MI, Non-fatal Stroke or Hospitalization for Unstable Angina
17	Bexagliflozin	PMID 31101403	7/6 (4.5%/3.9%)	Seven patients suffered fracture in drug group. Six patients suffered fracture in placebo group.	change in percent HbA1c at week 24	changes in body weight, sBP, albuminuria, and HbA1c stratified by CKD stage.
18		PMID 31161692/34292100	1/0 (0.05%/0.00%)	One patient suffered fracture in Bexagliflozin, no fracture in Sitagliptin group.	The non-inferiority of bexagliflozin to sitagliptin for change in HbA1c from baseline to week 24.	Changes from baseline to week 24 in FPG, BMI and SBP.
19		RCT (NCT02558296)		total complete 1046/531, **Femur fracture 3:0 (0.27:0.00) Hip fracture 2:1 (0.18:0.18)** Patella fracture 3:0 (0.27:0.00) Foot fracture 2:0 (0.18:0.00) Rib fracture 2:0 (0.18:0.00) Spinal fracture 2:0 (0.18:0.00) Compression fracture 0:1 (0.00:0.18) Fibula fracture 1:0 (0.18:0.00) Lower limb fracture 1:0 (0.18:0.00)	Change in HbA1c to Week 24	1.Change in HbA1c at Week 24 for Subjects Who Have Been Prescribed Insulin. 2.Change in Body Weight, SBP at week 48
20	LIK-066 (Licogliflozin)	RCT (NCT03320941)	1/15 (6.67%); 0/16 (0.00%); 0/30 (0.00%); 1/30 (3.33%); 0/33 (0.00%)	no fracture reported	Percentage Change in body weight at Week 12.	1.Responder Rates of Percentage Decrease in Body Weight at Week 12. 2.Percentage Change Body Weight, HbA1c, FPG, SBP, DBP, Uric Acid, Urine Albumin, Visceral Fat Area (VFA) at Week 12. 3.Change at Week 12 on Waist Circumference at Umbilical Level. 4.Percentage Change at Week 12 on Fasting Lipid Profile, reactive Protein (hsCRP).
21	Henagliflozin Proline/SHR3824	RCT (NCT04390295)		no fracture reported	Adjusted Mean Change in HbA1c Levels	1.Adjusted Mean Change in FBG. 2.The number of adverse events.
22	Remogliflozin etabonate	RCT (CTRI/2017/07/009121)		No fracture was reported.	Mean change from baseline in HbA1c levels at week 24.	Mean change in HbA1c at week 12, proportion of therapeutic glycemic response achievement (HbA1c<7%) at 24 weeks, proportion rescue medication requirement, mean change in FPG, PPG, bodyweight, fasting lipids, SBP and DBP at 12 and 24 weeks.

*Table 1 and **Table 2** correspond by column of No.

*The figures in column of Result are all percent. eg. 0.03:0.09 means 0.03%:0.09%.

CV, cardiovascular; MI, myocardial infarction; FBG, fasting blood glucose; SBP, Systolic Blood Pressure; DBP, diastolic blood pressure.

A summary of the clinical evidence of the meta-analysis results of SGLT-2is on bone fracture is shown in **Table 4** A systematic review and network meta-analysis compared the effects of anti-diabetic agents on fracture risk. The results indicated that empagliflozin and ertugliflozin may increase the risk of fracture, while dapagliflozin may show benefits (70). A study that included four SGLT-2is (dapagliflozin, empagliflozin, canagliflozin, and ertugliflozin) aimed to evaluate their safety in three chronic diseases: T2DM, chronic heart failure (CHF), and CKD. SGLT-2is showed increased trends of risk of fracture [risk ratio (RR) = 1.07, 95%CI = 0.99–1.16, *p* = 0.081] (71). The results of a meta-analysis of 38 RCTs showed fracture odds ratios (ORs) of canagliflozin, dapaglizin, and empagliflozin of 1.15, 0.68, and 0.93, respectively, compared to placebo (72). There was no increase in the risk of bone fracture observed among patients with T2DM treated with SGLT-2is compared with placebo. Another meta-analysis of 20 clinical studies also found similar results (73). A nationwide Medicare cohort of older patients with T2DM initiated on SGLT-2is did not show associations with an increased risk of fracture compared with the initiation of DPP-4i or GLP-1RA, with consistent results across categories of frailty, age, and insulin use (81). A meta-analysis of safety profiles comparing canagliflozin, empagliflozin, and tofogliflozin in Japanese patients with T2DM indicated that SGLT-2is were related to a similar risk of fracture to placebo (RR = 0.85, 95%CI = 0.20–3.61) (82). This is consistent with data on East Asian patients and 80% of Caucasian patients (72). However, a sub-analysis of the network meta-analysis showed that Asian populations exhibit a higher risk of fracture (82). Another study used a random effects model to estimate the RRs and summarized that the use of SGLT-2is (RR = 1.02, 95%CI = 0.91–1.16, *n* = 4) is not associated with the risk of fracture (74).

**Table 4:** Meta-analysis of the effect of SGLT-2 inhibitors on bone fracture.

Reference	Number of studies	Type of study	Patients included	Comparators	Fracture RR	95%CI	Consults
(70)	17	RCTs	221364	placebo	canagliflozin (RR 0.62; 0.13–3.08) dapagliflozin (RR 0.9; 0.16–5.14) empagliflozin (RR 1.19; 0.24–5.89) ertugliflozin (RR 2.47; 0.16–9.95)		SGLT2is did not modify the risk of fracture with statistically significant differences.
(71)	8	large RCTs	59692	placebo	1.07	0.99-1.16	SGLT2is showed the increased trends in the risks of fracture.
(72)	38	RCTs	30384	placebo	canagliflozin (OR 1.15; 0.71-1.88) dapagliflozin (OR 0.68; 0.37-1.25) empagliflozin (OR 0.93; 0.74-1.18)		Not support the harmful effect of SGLT2is on fractures.
(73)	20	RCTs	8286	placebo	SGLT-2is (0.67, 0.42-1.07) canagliflozin (0.66, 0.37-1.19) dapagliflozin (0.84, 0.22-3.18) empagliflozin (0.57, 0.20-1.59)		Increased risk of bone fracture among T2DM patients treated with SGLT2is compared with placebo was not observed.
(74)	18	14 cohort studies, 4 case-control studies	–	placebo or active comparators (DPP4i, GLP-1RA)	1.02	0.91-1.16	Cumulative real-world evidence does not support an association between the use of DPP-4i, GLP-1ra, or SGLT2i and the risk of fracture.
(75)	20	RCTs	129465	placebo	1.02	0.88-1.88	SGLT2is probably have no effect on bone fracture.
(76)	40	RCTs	32343	placebo	1.01	0.83-1.23	No detrimental effect of SGLT2is on fracture risk in patients with T2DM.
(77)	109	109 publications	112 randomized populations	placebo or active comparators	0.87	0.69-1.09	Current evidence from RCTs does not suggest an increased risk of harm with SGLT2is over placebo or active comparators.
(78)	13	RCTs	14618		--	--	The occurrence of bone fracture did not differ between SGLT-2i and placebo group.
(79)	30	RCTs	23372	placebo	0.86	0.70-1.06	SGLT2is do not increase risk of bone fracture compared with placebo in T2DM patients.
(80)	27	RCTs	20895	placebo	1.02	0.81-1.28	No increased risk for bone fracture was detected in T2DM patients treated with SGLT2is.

Based on the mechanism of SGLT-2is on bone mineral metabolism, they may cause secondary PTH increase, promote bone resorption, and cause bone microstructure damage, which are established fracture risks. Currently, among the main large-scale clinical trials that included dapagliflozin, empagliflozin, and canagliflozin, only canagliflozin in the CANVAS Study increased the risk of bone fracture and reduced BMD compared to placebo. Most of the participants in the CANVAS Study were with established cardiovascular disease, and these patients have a higher risk of fracture, which may lead to biased results. In the CANVAS-R study, which involved T2DM patients with albuminuric CKD or a reduced estimated glomerular filtration rate (eGFR), no significant differences in the rates of amputation or fracture were observed. In the same study, the participants may have had potential CKD–mineral and bone disorders (MBDs), which makes the bone and mineral metabolism more complex. In addition, the effect of canagliflozin, an SGLT-1 and SGLT-2 co-inhibitor, on BMD and bone fracture may be associated with the oversuppression of both SGLT-1 and SGLT-2, but other SGLT-2is only act on SGLT-2. Thus, the effect of another dual SGLT-1/SGLT-2 inhibitor, sotagliflozin, on bone fracture was expected.

According to meta-analysis evidence (70–74, 81, 82) (**Table 4**), most of the meta-analysis results indicated that the SGLT-2is did not increase the risk of bone fracture across categories of age, race, type of SGLT-2is, and underlying comorbidities. The fracture risk of SGLT-2is is controversial. Most clinical trials on SGLT-2is have focused on the primary endpoint of blood glucose control or cardiovascular renal outcomes and had short intervention and follow-up periods. Moreover, the negligible number of fractures may have caused statistical bias. To obtain firm evidence of the risk of fracture, BMD, and incidence of bone fracture, a longer follow-up time is needed. Furthermore, suitable participants, such as those with T2DM with established osteoporosis or osteopenia, and the inclusion and exclusion criteria should be designed to focus on parameters of bone fracture risk. Therefore, more large-scale clinical trials or real-world studies are needed to provide convincing evidence. In addition, SGLT-2is treatment may cause hypoglycemia, relative insufficiency of blood pressure and blood volume, and postural hypotension and may induce accidental falls and fractures (83). Based on its significant glucose-lowering effect and benefit on the cardiovascular renal outcomes and prognosis, SGLT-2is are an outstanding choice for T2DM. Full consideration of the advantages and disadvantages is necessary for T2DM patients with a high risk of falls and bone fracture, such as those who are aging, with weakness, osteoporosis, or osteopenia, those with previous fracture history, and those using other concomitant medications affecting fracture, among others. It is helpful to access BMD and fracture risk assessments when administering SGLT-2is to patients with a high risk of fracture.

## Conclusion

In conclusion, SGLT-2is affect mineral homeostasis and bone/mineral-regulating hormone by promoting the excretion of sodium and glucose. The effect of SGLT-2is on bone fracture is controversial. In general, the use of SGLT-2is did not show an association with increased risk of bone fracture. SGLT-2is reduce blood glucose and effective circulating volume, which may lead to the risk of falls and fractures. Assessment of BMD and fracture risk should be considered, especially for patients with a high risk of fracture.

## Author Contributions

BD and RL drafted the manuscript. BD designed and prepared the figures. JW, LC, ZW, and ZH provided helpful suggestions. YW conceived the study. LX designed the study and take responsibility for this study. All authors contributed to the article and approved the submitted version.

## Funding

This work was supported by a grant from the National Natural Science Foundation of China (no. 81600691) and a China Postdoctoral Science Foundation-funded project (no. 2018M640615). The content of the article has not been influenced by the sponsors.

## Conflict of Interest

The authors declare that the research was conducted in the absence of any commercial or financial relationships that could be construed as a potential conflict of interest.

## Publisher’s Note

All claims expressed in this article are solely those of the authors and do not necessarily represent those of their affiliated organizations, or those of the publisher, the editors and the reviewers. Any product that may be evaluated in this article, or claim that may be made by its manufacturer, is not guaranteed or endorsed by the publisher.

## Supplementary Material

Supplementary Figure 1 Flowchart of the identification of eligible literatures. To estimate the effect of SGLT2is on BMD and bone fracture risk in patients with T2DM, we comprehensively searched Pubmed, Medline, Embase, the Cochrane Database of Systematic Reviews and ClinicalTrials.gov to identify all eligible trials. We searched various articles including the mechanism of SGLT-2is affecting bone metabolism, relevant clinical trials, animal studies, *in vitro* experiments and so on. Reference lists of relevant articles were also performed to retrieve additional studies. Two researchers searched and screened the literatures independently. The terms used for the research included “sodium–glucose co-transporter 2 inhibitors”, “SGLT-2 inhibitor”, “dapagliflozin”, “canagliflozin”, “empagliflozin”, “ipragliflozin”, “tofogliflozin”, “luseogliflozin”, “ertugliflozin”, “sotagliflozin”, “janagliflozin”, “bexagliflozin”, “licogliflozin”, “henagliflozin”, “remogliflozin”, “tianaglilflozin”, “bone and mineral metabolism”, “bone turnover”, “bone fracture”, “type 2 diabetes mellitus”, “T2DM” with no language restrictions. We screened and focused on clinical trials and studies, or animal studies, meta-analysis and systemic reviews and so on. We excluded duplicate publications, case reports, ongoing trials without results, not available full-texts literatures and those not associated with mineral bone metabolism or fracture.

## Abbreviations

SGLT-2i, sodium-glucose co-transporter 2 inhibitor; PTH, parathyroid hormone; CKD, chronic kidney disease; T2DM, type 2 diabetes mellitus; GLUT, glucose transporters; Na, sodium; Ca, calcium; Mg, magnesium; Pi, phosphorus; BMD, bone mineral density; FGF23, fibroblast growth factor 23; IR, insulin resistance; RAAS, renin–angiotensin–aldosterone system
