# Supplementary figures and images for: The Extraglycemic Effect of SGLT-2is on Mineral and Bone Metabolism and Bone Fracture
Source: Front Endocrinol (Lausanne). 2022 Jul 7;13:918350. doi: 10.3389/fendo.2022.918350 (PMC9302585; doi:10.3389/fendo.2022.918350)

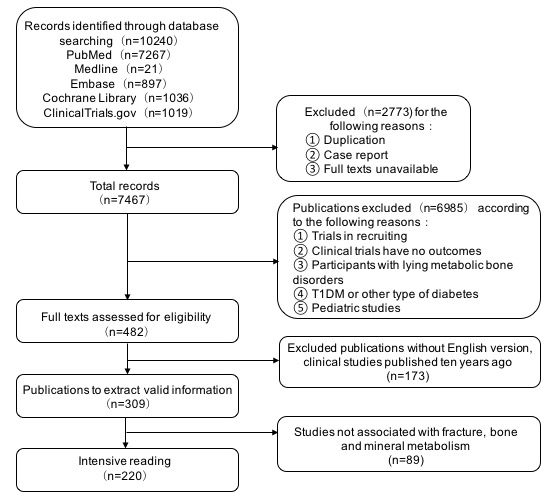

Supplement: Supplementary Figure 1 — Flowchart of the identification of eligible literatures. To estimate the effect of SGLT2is on BMD and bone fracture risk in patients with T2DM, we comprehensively searched Pubmed, Medline, Embase, the Cochrane Database of Systematic Reviews and ClinicalTrials.gov to identify all eligible trials. We searched various articles including the mechanism of SGLT-2is affecting bone metabolism, relevant clinical trials, animal studies, in vitro experiments and so on. Reference lists of relevant articles were also performed to retrieve additional studies. Two researchers searched and screened the literatures independently. The terms used for the research included “sodium–glucose co-transporter 2 inhibitors”, “SGLT-2 inhibitor”, “dapagliflozin”, “canagliflozin”, “empagliflozin”, “ipragliflozin”, “tofogliflozin”, “luseogliflozin”, “ertugliflozin”, “sotagliflozin”, “janagliflozin”, “bexagliflozin”, “licogliflozin”, “henagliflozin”, “remogliflozin”, “tianaglilflozin”, “bone and mineral metabolism”, “bone turnover”, “bone fracture”, “type 2 diabetes mellitus”, “T2DM” with no language restrictions. We screened and focused on clinical trials and studies, or animal studies, meta-analysis and systemic reviews and so on. We excluded duplicate publications, case reports, ongoing trials without results, not available full-texts literatures and those not associated with mineral bone metabolism or fracture. [file Image_1.jpeg]
